# Dose-Independent ADME Properties and Tentative Identification of Metabolites of α-Mangostin from *Garcinia mangostana* in Mice by Automated Microsampling and UPLC-MS/MS Methods

**DOI:** 10.1371/journal.pone.0131587

**Published:** 2015-07-15

**Authors:** Seung Yon Han, Byoung Hoon You, Yu Chul Kim, Young-Won Chin, Young Hee Choi

**Affiliations:** 1 College of Pharmacy and BK21 PLUS R-FIND Team, Dongguk University-Seoul, 32 Dongguk-lo, Ilsandong-gu, Goyang, Gyeonggi-do, 410–820, South Korea; 2 Discovery Research Center, C&C Research Laboratories, 2066 Seobu-lo, Suwon-si, Gyeonggi-do, 440–746, South Korea; University of Kentucky, UNITED STATES

## Abstract

The information about a marker compound's pharmacokinetics in herbal products including the characteristics of absorption, distribution, metabolism, excretion (ADME) is closely related to the efficacy/toxicity. Also dose range and administration route are critical factors to determine the ADME profiles. Since the supply of a sufficient amount of a marker compound in *in vivo* study is still difficult, pharmacokinetic investigations which overcome the limit of blood collection in mice are desirable. Thus, we have attempted to investigate concurrently the ADME and proposed metabolite identification of α-mangostin, a major constituent of mangosteen, *Garcinia mangostana* L, in mice with a wide dose range using an *in vitro* as well as *in vivo* automated micro-sampling system together. α-mangostin showed dose-proportional pharmacokinetics at intravenous doses of 5–20 mg/kg and oral doses of 10–100 mg/kg. The gastrointestinal absorption of α-mangostin was poor and the distribution of α-mangostin was relatively high in the liver, intestine, kidney, fat, and lung. α-mangostin was extensively metabolized in the liver and intestine. With regards to the formation of metabolites, the glucuronidated, bis-glucuronidated, dehydrogenated, hydrogenated, oxidized, and methylated α-mangostins were tentatively identified. We suggest that these dose-independent pharmacokinetic characteristics of α-mangostin in mice provide an important basis for preclinical applications of α-mangostin as well as mangosteen. In addition, these experimental methods can be applied to evaluate the pharmacokinetics of natural products in mice.

## Introduction

As the use of herbal products as adjuvant or alternative medicines has been increasing, the evaluation of the therapeutic outcomes of pharmacologically active compounds in herbal products is essential at the preclinical and clinical levels [1], [2]. In the prediction of therapeutic outcomes, including efficacy and safety, the pharmacokinetic characteristics of compounds with a wide dose ranges are fundamental [3], [4], [5], [6], [7] and the US FDA encourages to monitor the blood levels of active compounds in herbal products as far as is feasible [US FDA Guidance for industry botanical drug products]. In addition, tissue distribution and plasma protein binding values elucidate the delivery and specific affinity of compounds to target organs [8], [9], of which factors are affected by dose ranges [7], [10]. Together with the above characteristics, the identification of metabolites in major metabolic organs helps predict the metabolic pathways and identify possible accumulation of parent compounds and/or metabolites [11], [12], [13].

Recently, attention has been paid to mice due to the use of relatively small amounts of compounds required and various applications on disease models including transgenic or xenografted mice [11], [12], [14], [15]. An automated micro-sampling and validated analytical method accelerates the pharmacokinetics of herbal products in mice with the reproducible and accurate blood sampling using only <10 μL of blood at each time [11], [12], [16]. The validated analytical method using high-performance liquid chromatography tandem mass-spectrometry (HPLC-MS/MS) allows analyzing a parent compound and tentative metabolites with sufficient quantitation limits [11], [12], [14].

α-Mangostin (α-MG), a major xanthone derivative from *Garcinia mangostana* L. a tropical fruit, exhibits anti-metastatic, renal protective and anti-allergic properties [17], [18], [19], [20], [21], [22]. Although the pharmacokinetic evaluation of α-MG was conducted using the plasma of rat [23], [24], [25] and *in vitro* hepatic metabolism tools [26], it is necessary to gain insight into the ADME of α-MG in mice due to the different physiological and pathological states of mice and rats [27]. In particular, collecting blood from tail saphenous veins or by heart puncture causes stress, which influences the normative pharmacokinetics by reducing absorption, delaying gastric emptying time and altering the metabolism [28], [29] and as such, the collection of multiple blood samples from conscious and freely moving mice using a micro-blood sampling system could yield different pharmacokinetic results.

This is the first report to explore ADME properties in mice including tissue distribution, protein binding and tentative metabolites identification of α-MG with a wide dose range using a microsampling system. Simultaneous investigation of the preferential tissue distribution and metabolite identification of α-MG might be an efficient means to predict any correlations with pharmacological activity in certain organs as well as the pharmacokinetics of metabolites. This integrated tool, with a microsampling and analytical system, enables simultaneous ADME screening and metabolite identification in mice in a manner different from previous reports [30], [31], [32], [33].

## Materials and Methods

### Materials

α-MG was purified (> 96.0% purity) in the College of Pharmacy, Dongguk University (Seoul, South Korea) according to the previously reported protocol [34]. Docetaxel [internal standard (IS) for HPLC-MS/MS; > 99% purity] was supplied from Shin Poong Pharmaceutical Company, Ltd (Ansan, South Korea). Polyethylene glycol 400 (PEG 400) was obtained from the Showa Chemical Company (Tokyo, Japan). Dextran, the reduced form of β-nicotinamide adenine dinucleotide phosphate (NADPH; as a tetrasodium salt), uridine diphosphate glucuronic acid (UDPGA; as a trisodium salt) and Tris-buffer were purchased from Sigma-Aldrich (St. Louis, MO, USA). Methanol, acetonitrile, formic acid and water were purchased from Fisher Scientific (Seoul, South Korea). All other chemicals and reagents used were of analytical grade.

### Animals

The protocols for the animal studies were approved by the Institute of Laboratory Animal Resources of Dongguk University, Seoul, South Korea (# 2012–0674; July 25, 2012). Male Institute of Cancer Research (ICR) mice (8 weeks old, weighing 20–30 g) were purchased from the Charles River Company Korea (Orient, Seoul, South Korea). The mice were acclimated for one week before starting the study. Upon arrival, animals were randomized and housed at three per cage under strictly controlled environmental conditions (20–25°C and 48–52% relative humidity). A 12-hour light/dark cycle was used at an intensity of 150 to 300 Lux.

### Pharmacokinetic studies of α-MG

The surgical procedures were conducted under intramuscular injection anesthesia with 125 mg (1.5 mL)/kg of tiletamine HCl and zolazepam HCl mixture. The carotid artery (for intravenous and oral studies) and the jugular vein (for intravenous study) were cannulated using CX-2052S and CX-2022S catheters (BASi, West Lafayette, IN), respectively. After the mice awoke from anesthesia, the administration of α-MG and blood sampling were performed [11], [12], [31].

In intravenous study, α-MG (dissolved in PEG 400: distilled water = 6:4, v/v) at doses of 5, 10 and 20 mg (5 mL)/kg was administered through the jugular vein. The micro-sampling system (Culex BASi, West Lafayette, IN) was programmed to collect a 10 μL sample of blood into a micro-vial containing 50 μL of 12.5 units/mL heparinized saline. Blood loss due to blood sampling was replaced with equal volumes of heparinized saline. Blood samples were collected at 0, 1, 5, 15, 30, 60, 90, 120, 180, 240, 300 and 360 min after intravenous administration of the α-MG with virtually no blood loss. After centrifugation of each micro-vial, 50 μL of supernatant was collected. At the end of 24 h, each metabolic cage was rinsed with 5 mL of distilled water and the resulting fluid combined with the urine collected over the previous 24 h. At this time, each mouse was sacrificed by cervical dislocation, and then the entire gastrointestinal tract (including its contents and feces) was removed, transferred into a beaker that contained 10 mL of methanol (to facilitate the extraction of α-MG) and the gastrointestinal tract was cut into small pieces. After manual shaking and stirring, a 50 μL aliquot of the supernatant was collected from each beaker and stored.

α-MG (the same solution as used in the intravenous study) at doses of 10, 50 and 100 mg (10 mL)/kg was administered orally using a gastric gavage tube after overnight fasting with free access to water. A blood sample using the microsampling system was collected via the carotid artery at 0, 5, 15, 30, 60, 90, 120, 180, 240, 300 and 360 min after the oral administration of α-MG. Other procedures were similar to those in the intravenous study.

### Tissue distribution of α-MG

The surgical procedures and administration of α-MG were conducted according to a previously reported method [11], [12], [31]. At 30 and 180 min after intravenous and oral administration of α-MG at doses of 10 mg/kg, as much blood as possible was collected via the carotid artery and each mouse was then sacrificed by cervical dislocation. Each liver, spleen, stomach, small intestine, large intestine, mesentery, kidney, fat, muscle, heart, lung, and brain was excised, weighted and homogenized with a volume of distilled water at 4 times the each organ. After centrifugation for 10 min, the supernatant was collected and all samples stored at –80°C until required.

### Mouse plasma protein binding of α-MG using an equilibrium dialysis technique

A 250 μL of mouse plasma was dialyzed against 250 μL of isotonic Sørensen phosphate buffer with pH 7.4 containing 3% (w/v) dextran (‘the buffer’) in a dialysis cell using a Spectra/Por 4 membrane (molecular weight cutoff of 12000–14000; Spectrum Medical Industries). α-MG was spiked into the plasma compartment to produce an initial concentration of 1 or 20 μg/mL and all other procedures followed the reported method [31].

### Metabolism of α-MG in *in vitro* S9 fractions from various tissues

The procedures for using S9 fractions from various tissues were similar those already reported [31]. Each liver, stomach, small intestine, large intestine, lung, heart, kidney, fat, mesentery, muscle, and brain was excised after cervical dislocation (*n* = 6). Each tissue sample was rinsed with cold 0.9% NaCl-injectable solution, blotted dry with tissue paper and weighed. Then each tissue was homogenized with a volume of 0.25 M sucrose at 4 times of each tissue. Metabolic activity was initiated by adding a 100 μL aliquot of the 9000 × *g* supernatant fraction (S9) of each tissue to a 135 μL of Tris-buffer (pH 7.4), 5 μL of 0.9% NaCl-injectable solution with 1 or 20 μg/mL α-MG as a final concentration, 5 μL (1 mM) of NADPH and 5 μL (3.3 mM) of UDPGA. A 250 μL aliquot of acetonitrile (containing 1 μg/mL of the IS) was added after a 30 min incubation in a thermomixer (37°C and 500 opm) to terminate the enzyme activity. The amount of remaining α-MG in the S9 fraction of each tissue was determined using the HPLC-MS/MS analytical method.

### Determination of α-MG

The analytical method for measuring α-MG was modified from a previous report [12]. In sample preparation, 100 μL of acetonitrile containing 1 μg/mL IS was added to 50 μL of aliquot of the sample. After vortex-mixing and centrifugation (15000 × *g* for 10 min), 10 μL of the supernatant was directly injected into a reversed-phase C_18_ column (Cadenza CD-C18, 2 mm × 75 mm i.d., 3-μm particle size; Imtakt, USA) kept at 4°C until injection. The analytes were monitored using a API4000 triple quadrupole mass spectrometer (AB Sciex, Foster City, CA) equipped with a turbo ion-spray interface for electrospray ionization and operated in positive ion mode at 5500 V and 500°C at 50 L/min of nebulizing gas flow, 50 L/min of turbo ion-spray gas flow, 20 L/min of curtain gas flow, 5500 V of ring voltage and 5 Torr of collision gas (nitrogen) pressure.

The mass transitions for α-MG and IS were *m/z* 411.14 → 354.99 (collision energy, 23 eV) and 808.379 → 527.200 (15 eV), respectively, in the multiple reaction monitoring (MRM) mode with positive ionization. These analytes were separated on the column with an isocratic mobile phase consisting of 0.1% formic acid in water and 0.1% formic acid in acetonitrile (30: 70, v/v) at a flow rate of 0.35 mL/min. The quantitation limit of α-MG was 1 ng/mL. All analytical data were processed using the Analyst software (Version 1.5.1; Applied Biosystems).

### Tentative identification of metabolites from α-MG

Probable metabolites of α-MG were identified using the simultaneous full scan MS, MRM and MS/MS modes in a Waters UPLC-XEVO TQ system (Waters Corporation, Milford, USA) to confirm the structures of any metabolites. The *m/z* ratios of the metabolites in plasma, urine, feces, liver, small intestine and S9 fractions of the liver and small intestine samples were determined from full scans in positive and negative modes from *m/z* 100 to 900. Structural elucidation of the metabolites was based on the fragmentation patterns of parent ion from the MS/MS mode at a collision energy of 10 to 20 eV generating daughter ions. The mass transitions for α-MG was *m/z* 411.21 → 354.85 (collision energy of 20 eV and collision cell exit potential of 25 eV, respectively). The unknown masses were further analyzed in MS/MS (daughter scan) mode with the electrospray ionization interface used to generate positive and negative ions at a capillary voltage of 3.0 kV, source temperature of 350°C and desolvation gas temperature of 650°C. The MRM methods for identification of possible metabolites generated by phase I and/or II reactions were included.

These compounds were separated on a reversed-phase C_18_ column (XSELECT CSH, 2.1 mm × 100 mm i.d., 1.7-μm particle size; Waters, Ireland) with a flow rate of 0.35 mL/min. The mobile phase composition was started at 95: 5 (v/v) of distilled water containing 0.1% formic acid (A) and acetonitrile (B) and gradually changed to 35: 65 (v/v) for 30 min, and then switched back to 95: 5 (v/v) for 40 min.

### Pharmacokinetic analysis

Standard methods [35] were used to calculate the following pharmacokinetic parameters using a non-compartmental analysis (WinNonlin 2.1; Pharmasight Corp., Mountain View, CA). The extent of absolute oral bioavailability (*F*) was calculated by dividing the AUC_oral_/AUC_iv_ at the same dose. The peak plasma concentration (*C*_max_) and time to reach *C*_max_ (*T*_max_) were read directly from the extrapolated data.

### Statistical analysis

A *P* value < 0.05 was deemed to be statistically significant using a student *t*-test between the two means for the unpaired data, or a Tukey’s multiple range test from the Social Package of Statistical Sciences (SPSS) *posteriori* analysis of variance (ANOVA) among the three or four means for the unpaired data. All data are expressed as mean ± standard deviations excepting the median (ranges) used for *T*_max_.

## Results

### Pharmacokinetic studies of α-MG

**Fig 1:**
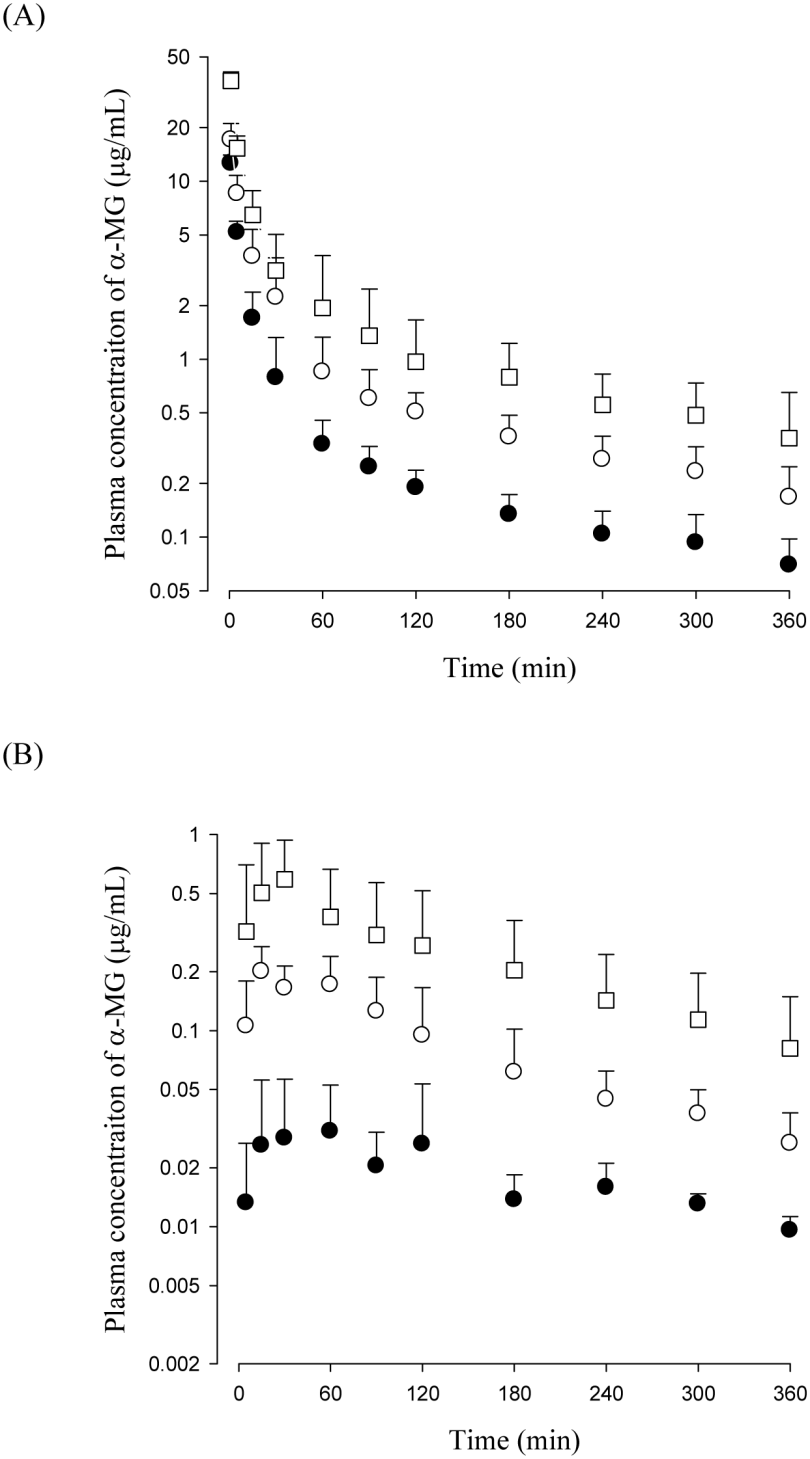
Mean plasma concentration of α-MG after intravenous administration of α-MG at doses of 5 (●), 10 (○) and 20 (□) mg/kg to mice (A). Also plasma concentration of α-MG after oral administration of α-MG at doses of 10 (●), 50 (○) and 100 (□) mg/kg to mice (B). Bars represent SDs.

The mean plasma concentration—time profiles of α-MG after the intravenous (5, 10 and 20 mg/kg) and oral (10, 50 and 100 mg/kg) administration of α-MG to mice are shown in Fig 1 and the relevant pharmacokinetic parameters are listed in Table 1. Note that the dose-normalized (based on 1 mg/kg of α-MG) *AUC values* of α-MG at doses of 5–20 mg/kg were not significantly difference among the mice. Similarly, no statistically significant difference was perceived in other parameters including the terminal half-life, mean residence time (MRT), apparent volume of distribution at steady state (*V*_ss_), total body clearance (CL), renal clearance (CL_R_), non-renal clearance (CL_NR_), and percentage of the intravenous dose of α-MG excreted in the urine as unchanged α-MG (*A*e_0–24 h_), and percentage of the intravenous dose of α-MG remaining in the gastrointestinal tract (including its contents and feces) at 24 h as unchanged α-MG (*GI*_24 h_). These results indicate that α-MG exhibits dose-independent (linear) pharmacokinetics at intravenous doses of 5–20 mg/kg in mice.

**Table 1:** Mean (± SD) plasma concentration of α-MG after intravenous and oral administration of α-MG to mice.

Parameters Intravenous	5 mg/kg (*n* = 9)	10 mg/kg (*n* = 8)	20 mg/kg (*n* = 8)
Body weight (g)	30.9 ± 5.67	36.4 ± 6.87	33.3 ± 3.46
AUC (μg min/mL)^**a**^	169 ± 42.7	340 ± 99.4	633 ± 226
Normalized AUC (μg min/mL)^**b**^	33.8 ± 8.53	32.1 ± 8.91	31.7 ± 11.3
Terminal half-life (min)	226 ± 34.3	219 ± 73.8	173 ± 71.9
MRT (min)	145 ± 27.7	160 ± 67.0	144 ± 68.5
CL (mL/min/kg)	31.4 ± 7.28	31.3 ± 7.95	34.7 ± 12.4
CL_R_ (mL/min/kg)	0.283 ±0.194	0.205 ± 0.150	0.141 ± 0.0801
CL_NR_ (mL/min/kg)	31.1 ± 7.14	31.1 ± 7.83	34.6 ± 12.3
*V*_ss_ (mL/kg)	4304 ± 1005	4270 ± 1974	4500 ± 1381
*Ae*_0–24 h_ (% of dose)	0.839 ± 0.525	0.592 ± 0.394	0.403 ± 0.163
*GI*_24 h_ (% of dose)	3.44 ± 1.16	2.28 ± 1.96	3.42 ± 1.73
Parameters	10 mg/kg (*n* = 8)	50 mg/kg (*n* = 7)	100 mg/kg (*n* = 9)
Oral			
Body weight (g)	25.0 ± 0.310	25.3 ± 1.89	27.9 ± 3.62
AUC (μg min/mL)^**a**^	7.80 ± 2.58	37.3 ± 10.5	83.2 ± 23.8
Normalized AUC (μg min/mL)^**b**^	0.861 ± 0.258	0.746 ± 0.209	0.831 ± 0.274
Terminal half-life (min)	177 ± 71.1	197 ± 81.0	151 ± 49.3
*C*_max_ (μg/mL)^**a**^	0.0403 ± 0.0307	0.242 ± 0.0350	0.709 ± 0.397
Normalized *C*_max_ (μg/mL)^**b**^	0.00403 ± 0.00306	0.00484 ± 0.000698	0.00708 ± 0.00396
*T*_max_ (min)^**c**^	60 (15–120)	30 (15–60)	60 (15–360)
CL_R_ (mL/min/kg)	0.535 ± 0.309	0.547 ± 0.309	0.418 ± 0.229
*Ae*_0–24 h_ (% of dose)	0.0255 ± 0.0107	0.0315 ± 0.0241	0.0270 ± 0.0113
*GI*_24 h_ (% of dose)	43.5 ± 10.6	40.2 ± 11.6	41.2 ± 4.51
*F* (%)	2.29		

All values were statistically analyzed and all of them were not statistically different (p < 0.05) except AUC and C_max_ values among three doses.

^**a**^ Statistically different (p < 0.05) among three doses.

^b^ Normalized values based on 1 mg/kg were not statistically different among three doses.

^c^ Median (ranges) for *T*_max_.

After oral administration of α-MG, α-MG was detected in the plasma from the first blood sampling time point (5 min), indicating rapid gastrointestinal absorption of α-MG. The dose-normalized (based on 1 mg/kg of α-MG) *AUC values of* α-MG at oral doses of 10–100 mg/kg indicated that α-MG has dose-independent (linear) pharmacokinetics at oral doses of 10–100 mg/kg in mice.

### Tissue distribution of α-MG

**Fig 2:**
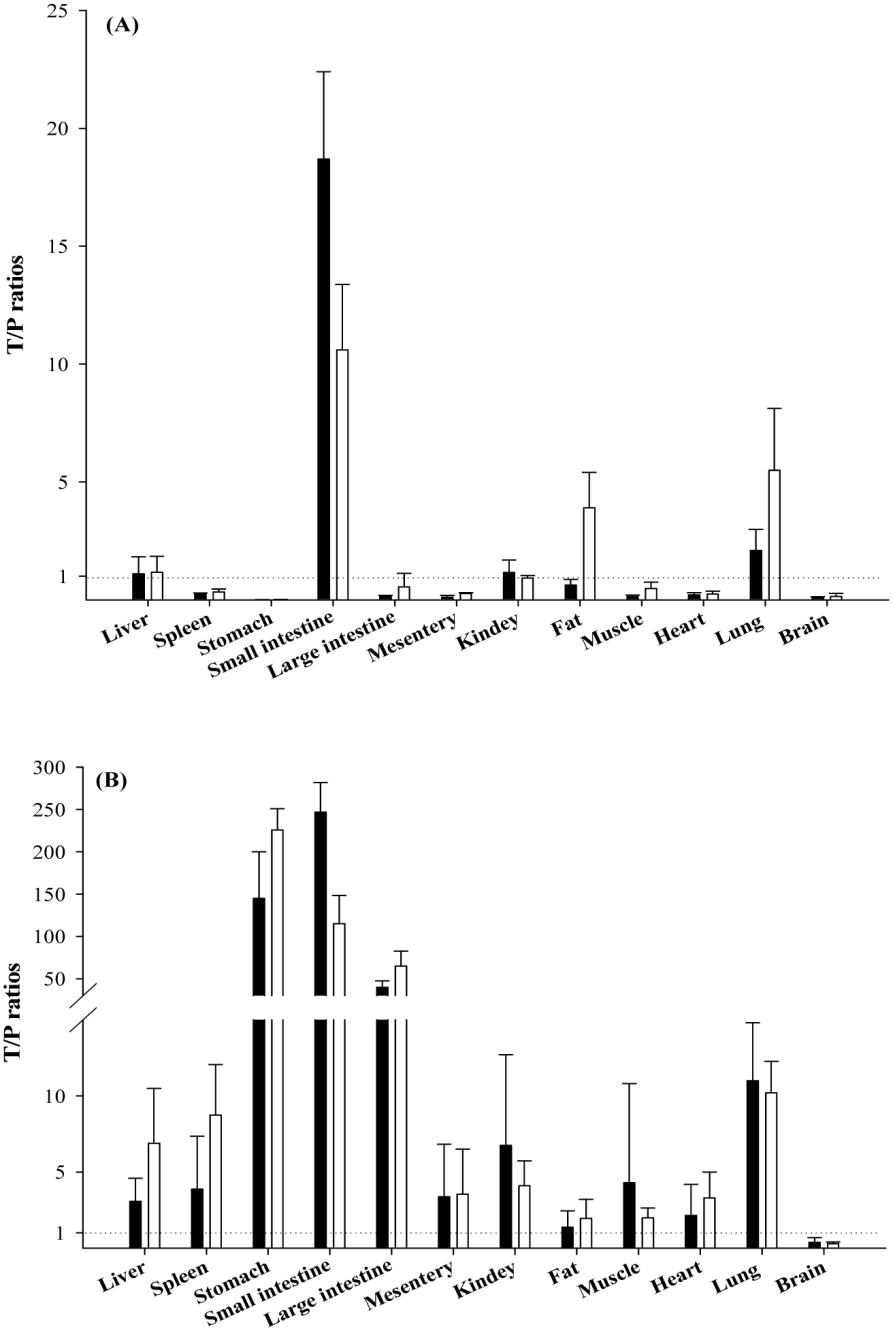
Mean T/P ratios of α-MG from various tissues at 30 (■) and 180 (□) min after intravenous (A) and oral (B) administration of α-MG at a dose of 10 mg/kg to mice. Bars represent SDs.

The degree of α-MG distributed to the tissues at 30 and 180 min after intravenous and oral administration of α-MG at a dose of 10 mg/kg are listed in Fig 2. The tissue-to-plasma (T/P) ratios of α-MG administered intravenously were greater than unity in the liver, small intestine and lung (at 30 and 180 min), kidney (at 30 min) and large intestine and fat (at 180 min). Likewise, the T/P ratios of α-MG administered orally were greater than unity for all tissues studied except the brain (at 30 and 180 min). These results indicate that the distribution of α-MG at a dose of 10 mg/kg to mouse tissues is substantial.

### Mouse plasma protein binding of α-MG using an equilibrium dialysis technique

The protein binding value of α-MG at a concentration of 1 or 20 μg/mL to fresh mouse plasma was 91.8 ± 13.5 or 89.3 ± 11.6%, respectively.

### Metabolism of α-MG in *in vitro* S9 fractions from various tissues

**Fig 3:**
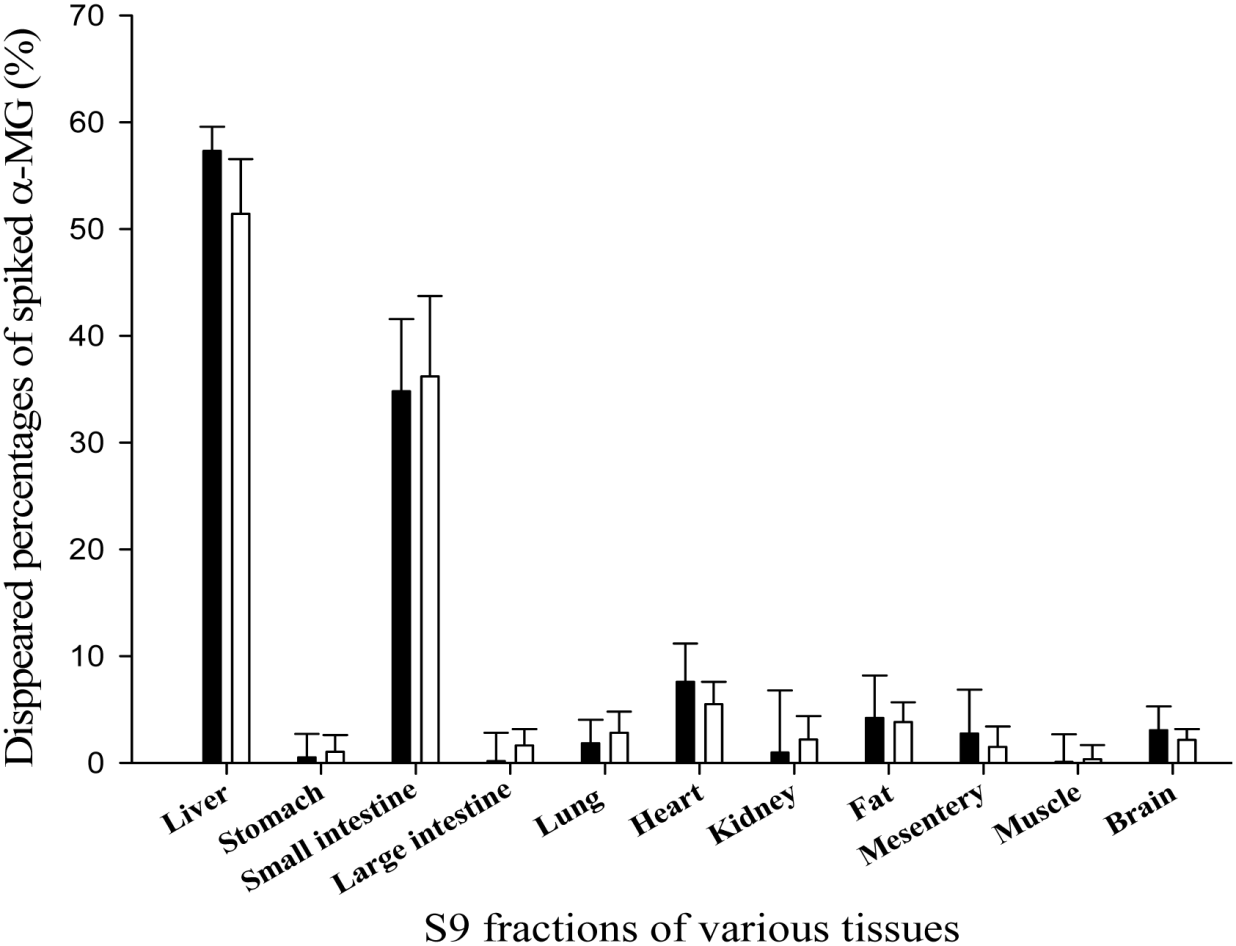
Mean values for the disappearance of α-MG after spiking 1 (■) or 20 (□) μg/mL of α-MG into S9 fractions of various tissues from mice. Bars represent SDs.

The values for the disappearance of α-MG at 1 or 20 μg/mL *in S9 fractions from various mouse tissues* are listed in Fig 3. In the S9 fractions of the liver and small intestine, 57.3 and 34.8% of the spiked 1 μg/mL of α-MG had disappeared (mainly metabolized), respectively. The corresponding values at 20 μg/mL of α-MG were 51.4 and 36.2% in liver and small intestine, respectively. However, other tissues studied had almost negligible metabolic activities for both spiked 1 and 20 μg/mL of α-MG.

### Tentative Identification of Metabolites from α-MG

**Fig 4:**
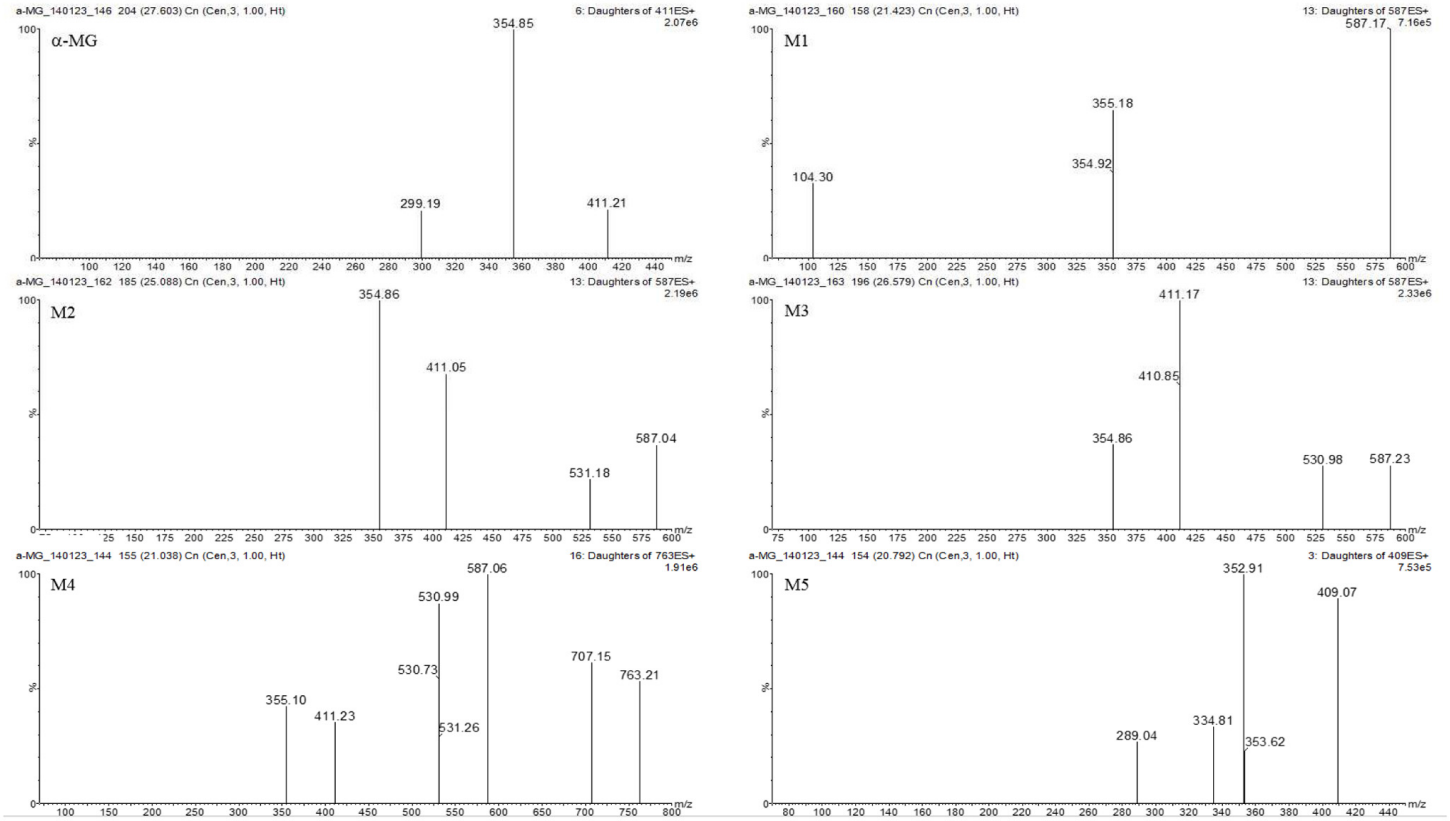
Spectrums of α-MG and its tentative metabolites (M1–M5) detected in mice’s plasma, urine, feces, liver and small intestine after intravenous and oral administration of α-MG and S9 fractions of the liver and small intestine after 30 min incubation.

**Fig 5:**
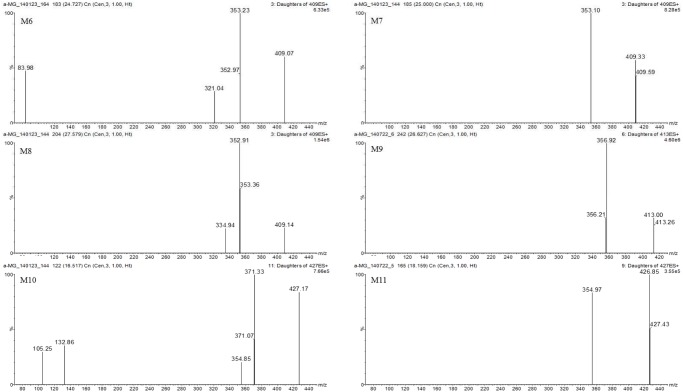
Spectrums of tentative metabolites (M6–M11) detected in mice’s plasma, urine, feces, liver and small intestine after intravenous and oral administration of α-MG and S9 fractions of the liver and small intestine after 30 min incubation.

**Fig 6:**
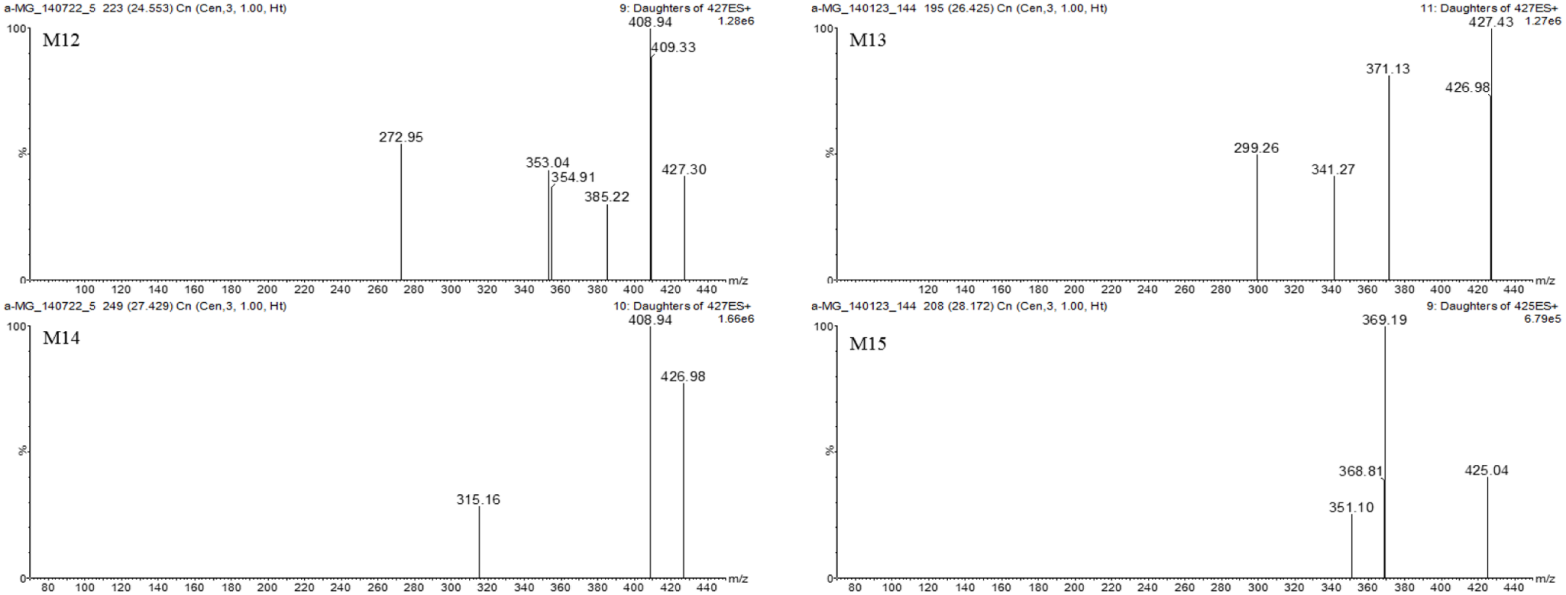
Spectrums of tentative metabolites (M12–M15) detected in mice’s plasma, urine, feces, liver and small intestine after intravenous and oral administration of α-MG and S9 fractions of the liver and small intestine after 30 min incubation.

The tentative identification of metabolites was based on different retention times, and the *m/z* ratios and fragment ions identified by LC-MS and MS/MS [36]. The MS/MS spectra of the proposed metabolites are shown in Figs 4–6 and the predicted structures of the metabolites are given in Table 2. M1–M3 (*m/z* 587.17, 587.04, and 587.23 with RT of 21.4, 25.1, and 26.6 min) were 176 Da higher than the [M+H]^+^ signal of α-MG (*m/z* 411.14), indicating that α-MG was metabolized via glucuronide conjugation. M4 (*m/z* 763.21, RT 21.0 min) was 352 Da higher than that of α-MG by bis-glucuronide conjugation. From the ions at the [M+H]^+^ signals of M5–M8, the m/z values of M5 (*m/z* 409.07, RT 20.8 min) and M6–M8 (*m/z* 409.07, 409.33, and 409.14 with RT of 24.0, 25.0, and 27.6 min) were 2 Da lower than that of α-MG, implying the formation of dehydrogenated metabolites from α-MG. The M9 (*m/z* 413.00, RT 26.6 min) might have been metabolized by a hydrogenation reaction. The *m/z* values of M10 and M11 (*m/z* 427.17 and 426.85 with RT 16.5 and 18.2 min) and M12–M14 (*m/z* 427.30, 427.43, 426.98 and 427.27 with RT 24.6, 26.4, and 27.4 min) were possibly formed via oxidation through adding O (16 Da) to α-MG. The 14 Da higher *m/z* of M15 (*m/z* 425.04, RT 28.2 min) than that of α-MG indicates that α-MG was metabolized by methylation. Here, the *m/z* of precursor and product ions of the possible metabolites were as follows: metabolites from glucuronide conjugation (*m/z* 587.3 → 531.1 or 355.1), bis-glucuronide conjugation (*m/z* 763.3 → 707.1 or 355.1), dehydrogenation (*m/z* 409.2 → 353.0), hydrogenation (*m/z* 413.3 → 357.1), oxidation (*m/z* 427.2 → 371.1 or 355.1), and methylation (*m/z* 425.3 → 369.1).

**Table 2:** Proposed metabolic reactions and metabolites in mice’s plasma, urine, feces, liver and small intestine after intravenous and oral administration of α-MG and S9 fractions of mice’s liver and small intestine after 30-min incubation.

Metabolite ([M + H]^+1^)	Fragment ions	RT^a^ (min)	Mass offset (Da)	Probable metabolic reaction	Source
α-MG (411.21)	354.85, 299.19	27.6		parent form	plasma, urine, feces, liver, small intestine, S9 fractions of liver and small intestine
M1 (587.17)	355.18, 104.30	21.4	176.034	glucuronide conjugation	plasma, urine, feces, liver, small intestine, S9 fractions of liver and small intestine
M2 (587.04)	531.18, 411.05, 354.86	25.1	176.034	glucuronide conjugation	plasma, urine, feces, liver, small intestine, S9 fractions of liver and small intestine
M3 (587.23)	530.98, 411.17, 354.86	26.6	176.034	glucuronide conjugation	plasma, urine, feces, liver, small intestine, S9 fractions of liver and small intestine
M4 (763.21)	707.15, 587.06, 530.99, 411.23, 355.10	21.0	352.068	bis-glucuronidation conjugation	plasma, feces, liver, small intestine
M5 (409.07)	352.91, 334.81, 289.04	20.8	-2.016	dehydrogenation	urine, feces, liver, small intestine
M6 (409.07)	353.23, 321.04, 83.98	24.7	-2.016	dehydrogenation	urine, feces, liver, small intestine
M7 (409.33)	353.10	25.0	-2.016	dehydrogenation	urine, feces, liver, small intestine
M8 (409.14)	352.91, 334.94	27.6	-2.016	dehydrogenation	urine, feces, liver, small intestine
M9 (413.00)	356.92	26.6	2.016	hydrogenation	urine
M10 (427.17)	371.33, 354.85, 132.86, 105.25	16.5	15.995	oxidation	liver, small intestine, feces
M11 (426.85)	354.97	18.2	15.995	oxidation	liver, small intestine, feces
M12 (427.30)	408.94, 385.22, 354.91, 353.04, 272.95	24.6	15.995	oxidation	liver, small intestine, feces
M13 (427.43)	371.13, 341.27, 299.26	26.4	15.995	oxidation	liver, small intestine, feces
M14 (426.98)	408.94, 315.16	27.4	15.995	oxidation	liver, small intestine, feces
M15 (425.04)	369.19, 351.10	28.2	14.016	methylation	liver, small intestine, feces

^a^RT: retention time.

## Discussion

The pharmacokinetic parameters of α-MG are useful to describe and predict information related to its efficacy and toxicity, including blood and tissue levels, determination of optimum dosage regimens, and correlation of drug concentration with pharmacological or toxicological activity [37]. All values on the Table 1 were statistically analyzed and all of them were not statistically different except AUC and C_max_ values among three doses. The intravenous normalized AUC of α-MG at doses of 5–20 mg/kg were not significantly different and oral normalized AUC and C_max_ values of α-MG at doses of 10–100 mg/kg were also not significantly different, indicating that α-MG showed dose-independent pharmacokinetics at these dosage ranges in mice (Table 1). After intravenous administration of α-MG at doses of 5, 10 and 20 mg/kg to mice, the *A*e_0–24 h_ values of α-MG were less than 0.839% of the intravenous dose (Table 1), suggesting that almost all intravenous α-MG is eliminated via a non-renal route (*CL*_NR_). The contribution of the gastrointestinal (including the biliary) excretion of α-MG to the *CL*_NR_ was almost negligible because the *GI*_24h_ values of α-MG were less than 3.44% of the doses (Table 1). Moreover, stable α-MG in various buffer solutions (having pHs ranging from 2 to 13; unpublished data) suggested that chemical and enzymatic degradation of α-MG did not seem to occur. Thus, the *CL*_NR_ of α-MG listed in Table 1 could represent the metabolic clearance of the drug.

The slower CL of α-MG (31.3–34.7 mL/min/kg; Table 1) than cardiac output (8 mL/min/0.02 kg) based on the plasma data (using hematocrit of 0.45 in mice; [27], [38]) indicated that α-MG in linear pharmacokinetic ranges can be eliminated by systemic metabolism in mice. This was proved by *in vitro* metabolism studies which showed that α-MG at spiked 1 and 20 μg/mL was metabolized the liver and small intestine (Fig 3) and the metabolic activity in liver and small intestine were similar even at spiked 1 and 20 μg/mL of α-MG. In addition, the CL_R_s of α-MG were estimated based on free (unbound to plasma proteins) fractions in plasma (CL_R, fu_); the CL_R, fu_ value thus estimated was 1.72–3.44 mL/min/kg. This value was considerably slower than the glomerular filtration rate of 14.0 mL/min/kg in mice (based on creatinine clearance; [27], [38]), suggesting that α-MG is excreted into urine predominantly via glomerular filtration in mice.

The *F* values of α-MG, only 2.29% of oral α-MG at 10 mg/kg, were low (Table 1). To find whether the poor gastrointestinal absorption of α-MG caused this low *F*, the ‘true’ fraction of the oral dose of α-MG unabsorbed (‘*F*_unabs_’) was calculated based on the reported equation assuming linear pharmacokinetics [39]. The estimated ‘*F*_unabs_’ value of 0.434 indicated that the poor gastrointestinal absorption of α-MG might be one of the main reasons for the low *F* of α-MG. The low absorption of α-MG in the Caco2 cell line [40] and α-MG as a substrate of P-glycoprotein in MDCK-mdr1 cell line (our unpublished data) supports the poor gastrointestinal absorption of α-MG in the *in vivo* system. Also the considerable degree of metabolism observed in the liver and intestine is another reason for the low *F* of α-MG.

**Fig 7:**
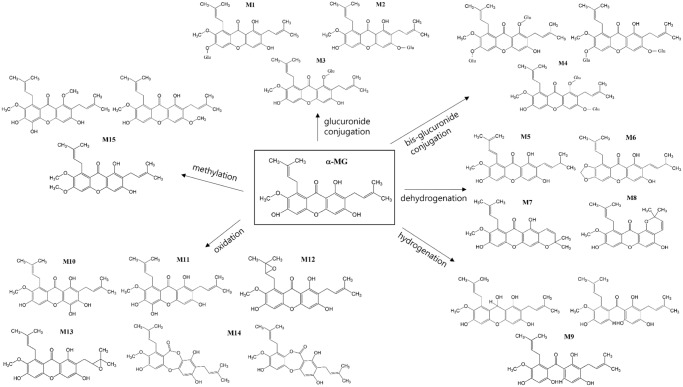
Proposed structures and metabolic pathways of metabolites in mice’s plasma, urine, feces, liver and small intestine after intravenous and oral administration of α-MG and S9 fractions of the liver and small intestine after 30 min incubation.

In aspect of extensive metabolism of α-MG, the chemical structures of the metabolites from α-MG were elucidated by LC-MS and MS/MS modes providing the [M+H]^+^ of α-MG, parent compounds and daughter ions thus giving more structural information. The components that presented at high concentrations were assumed to be the parent compounds [41] and the tentative metabolic pathways of α-MG were as follows: glucuronide conjugation (*m/z* 587.3 → 531.1 or 355.1), bis-glucuronide conjugation (*m/z* 763.3 → 707.1 or 355.1), dehydrogenation (*m/z* 409.2 → 353.0), hydrogenation (*m/z* 413.3 → 357.1), oxidation (*m/z* 427.2 → 371.1 or 355.1), and methylation (*m/z* 425.3 → 369.1). The probable metabolites suggest that α-MG was metabolized via phase I/II reactions. The enzymatic reactions such as glucuronide and bis-glucuronide conjugation via UGTs and oxidation, methylation, hydrogenation and dehydrogenation via CYPs, respectively [42], [43], supported the metabolic pathways of α-MG shown in Fig 7 [44].

The observed MS/MS fragments of the proposed metabolites were based on those of α-MG, *m/z* of 354.99 and 299.13, because the mass transition of the daughter ion at 354.99 was from [M+H]^+^–R (H_2_C = CCH_3_CH_3_) and 299.13 from [M+H]^+^–2R. Considering the RT shift of α-MG, the polarities of the metabolites generated by phase I/II reactions, excepting methylation, were increased because all RT values were shifted to the left of α-MG. The reduced hydrophilicity of methylated metabolite was also observed in another report [36]. Among dehydrogenated metabolites (M5–M8), the cyclized structures M6–M8 (Fig 7) were proposed from the previous report that cyclized metabolite of α-MG was made by microbial transformation [45].

The polarity of M5 seemed to be higher than other metabolites (M6-M8) and we predicted that the RT of M5 was 20.8 min, the shortest RT among M5–M8. Furthermore, M10 and M11 seemed to show the shorter RT than those of M12–M14, because M12 and M13 were formed by epoxidation and M14 was formed by oxidation and ring formation, whereas the hydroxylation was related to M10 and M11. In oxidation, the relative retention shift by epoxidation and ring formation with oxidation were closer to 1 than hydroxylation, which indicated that the hydroxylated metabolite was more polar than metabolites formed via epoxidation or ring formation [36]. The metabolites formed by glucuronide and bis-glucuronide conjugation were circulated into the blood. Also the metabolites formed by dehydrogenation, oxidation, hydrogenation, and methylation were observed at low levels and were probably eliminated into urine and feces.

In tissue distribution study, after intravenous administration of α-MG at a dose of 10 mg/kg, the high T/P ratios in the liver, small intestine, fat and/or lung were higher than unity (T/P = 1), suggesting that α-MG was highly distributed to these tissues with high affinities. Especially in the fat and lung, the T/P ratios showed a marked increase at 180 min, indicating that α-MG distributed slowly to these tissues. After oral administration of α-MG at a dose of 10 mg/kg, the T/P ratios were above unity in all tissues except the brain, indicating that α-MG distributed well to most tissues. After intravenous and oral administration of α-MG at doses of 5 mg/kg [31] and 20 mg/kg (our unpublished data) to mice, the tendencies of tissue distribution of α-MG were similar to those at 10 mg/kg in this study, indicating that α-MG showed the dose-independent tissue distribution from 5 to 20 mg/kg to mice. The high T/P ratios of α-MG indicate that α-MG might show pharmacological or toxicological activity in targeted tissues even if the plasma concentration was under the therapeutic range or the *F* was low. For example, the therapeutic potential of α-MG in a mouse model of allergic asthma might be in part explained by the high T/P of α-MG in the lung in spite of the low plasma concentration of α-MG [46].

In conclusion, α-MG showed dose-independent pharmacokinetics at intravenous (5–20 mg/kg) and oral (10–100 mg/kg) doses. The *F* of α-MG was low, 2.29%, and this could be due to the poor gastrointestinal absorption and/or extensive metabolism of α-MG in the liver and small intestine. Also the distribution and affinities of α-MG to the liver, small intestine, kidney, fat and lung were high after intravenous administration, while those of α-MG were high in most tissues except the brain after oral administration. Possible metabolites of α-MG via glucuronide conjugation, bis-glucuronide conjugation, dehydrogenation, hydrogenation, oxidation, and methylation have been proposed. These findings may be useful in understanding the efficacy and toxicity of α-MG in various preclinical models with specific diseases for the design of adjunctive therapies using α-MG.
